# Impact of treatment in long-term survival patients with follicular lymphoma: A Spanish Lymphoma Oncology Group registry

**DOI:** 10.1371/journal.pone.0177204

**Published:** 2017-05-11

**Authors:** Mariano Provencio, Pilar Sabín, Jose Gomez-Codina, Maria Torrente, Virginia Calvo, Marta Llanos, Josep Gumá, Cristina Quero, Ana Blasco, Miguel Angel Cruz, David Aguiar, Francisco García-Arroyo, Javier Lavernia, Natividad Martinez, Manuel Morales, Alvaro Saez-Cusi, Delvys Rodriguez, Luis de la Cruz, Jose Javier Sanchez, Antonio Rueda, on behalf of GOTEL (Spanish Lymphoma Oncology Group)

**Affiliations:** 1 Department of Medical Oncology, Hospital Universitario Puerta de Hierro-Majadahonda, Madrid, Spain; 2 Department of Medical Oncology, Hospital Universitario Gregorio Marañón, Madrid, Spain; 3 Department of Medical Oncology, Hospital Universitario La Fe, Valencia, Spain; 4 Department of Medical Oncology, Hospital Universitario de Canarias, Tenerife, Spain; 5 Department of Medical Oncology, Hospital Universitario San Joan de Reus, Tarragona, Spain; 6 Department of Medical Oncology, Hospital Universitario Virgen de la Victoria, Málaga, Spain; 7 Department of Medical Oncology, Hospital General Universitario, Valencia, Spain; 8 Department of Medical Oncology, Hospital Virgen de la Salud, Toledo, Spain; 9 Department of Medical Oncology, Hospital Universitario de Gran Canaria Doctor Negrín, Las Palmas, Spain; 10 Department of Medical Oncology, Complejo Hospitalario de Pontevedra, Pontevedra, Spain; 11 Department of Oncology, Instituto Valenciano de Oncología, Valencia, Spain; 12 Department of Medical Oncology, Hospital General Universitario de Elche, Alicante, Spain; 13 Department of Medical Oncology, Hospital Universitario Nuestra Señora de Candelaria, Santa Cruz de Tenerife, Spain; 14 Department of Medical Oncology, Hospital Clínico Universitario Lozano Blesa, Zaragoza, Spain; 15 Department of Medical Oncology, Hospital Universitario Insular de Gran Canaria, Las Palmas, Spain; 16 Department of Medical Oncology, Hospital Universitario Virgen de la Macarena, Sevilla, Spain; 17 Universidad Autónoma de Nuevo León, Monterrey, Mexico; 18 Department of Medical Oncology, Hospital Costa del Sol, Marbella, Spain; Universidad de Navarra, SPAIN

## Abstract

**Background:**

Follicular lymphoma is the second most common non-Hodgkin lymphoma in the United States and Europe. However, most of the prospective randomized studies have very little follow-up compared to the long natural history of the disease. The primary aim of this study was to investigate the long-term survival of our series of patients with follicular lymphoma.

**Patients and methods:**

A total of 1074 patients with newly diagnosed FL were enrolled. Patients diagnosed were prospectively enrolled from 1980 to 2013.

**Results:**

Median follow-up was 54.9 months and median overall survival is over 20 years in our series. We analyzed the patients who are still alive beyond 10 years from diagnosis in order to fully assess the prognostic factors that condition this group. Out of 166 patients who are still alive after more than 10 years of follow-up, 118 of them (73%) are free of evident clinical disease. Variables significantly associated with survival at 10 years were stage < II (*p* <0.03), age < 60 years (*p* <0.0001), low FLIPI (*p* <0.002), normal β2 microglobulin (*p* <0.005), no B symptoms upon diagnosis (*p* <0.02), Performance Status 0–1 (*p* <0.03) and treatment with anthracyclines and rituximab (*p* <0.001), or rituximab (*p* <0.0001).

**Conclusions:**

A longer follow-up and a large series demonstrated a substantial population of patients with follicular lymphoma free of disease for more than 10 years.

## Introduction

Follicular lymphomas (FL) are the second most common non-Hodgkin lymphoma (NHL) in Western Europe [1], accounting for approximately 20 percent to 30 percent of all NHLs. In the series from The Non-Hodgkin´s Lymphoma Classification Project they represent 22% [2, 3], and 22–40% of the NHL [4, 5] according to the WHO classification.

The annual incidence of this disease has increased rapidly in the last decades, from 2–3 cases in a population of 100,000 in 1950 to 5.7 cases/100,000 in 2009. The prevalence is of approximately 40/100.000 [6]. The incidence increases with age. Most cases occur in adults over 50 years old and the elderly. They are rare in the third and fourth decades and exceptional in children and adolescents. In Spain, between 3,000 and 5,000 new cases of follicular lymphoma are diagnosed each year. It is the second most common tumor of lymphoid lineage.

Despite recent improvements in survival, FL remains an incurable disease. About 80% of patients with FL are diagnosed in stages III / IV. The median of the overall survival (OS) is lengthy (about 10 years) and OS rate up to five years over 75% [7]. Additionally, transformation to more aggressive histological forms, usually diffuse large B-cell lymphoma (DLBCL), may also occur, which implies, in general, very poor prognosis [8]. The risk of transformation appears to be independent of the type of treatment used (or lack thereof) [9]. In autopsy series, most cases (95%) show some evidence of transformation [10].

The course of the disease can be highly variable [11]. This is a difficult task in such an uncommon disorder with a prolonged natural history, and almost impossible when complex treatments such as bone marrow transplantation are concerned. In order to facilitate clinical studies, a number of outcome biomarkers have been proposed, although none of them has a validated correlation with survival.

The Follicular Lymphoma International Prognostic Index (FLIPI), has been proven to have a better discriminatory power in assessing patient prognosis and seems to produce a more even patient distribution among different risk groups compared to the International Prognostic Index (IPI). The FLIPI is currently used for defining individual risk of death and the tumor grade [12, 13, 14]. More recently, FLIPI2 [15] was developed as a new model for prognostic definition of patients with FL. It will best fit the current reality of the problem, as it has been developed with patients treated with immunotherapy and discriminates in groups according to a progression-free interval of disease, which is a more appropriate variable for FL.

Patients usually have prolonged survival, with medians that can reach or exceed 10 years. They have high response rates to different treatments, although responses are followed by sequent relapses with a shrinking time interval. Despite this high chemosensitivity, historically, nor OS or disease free survival could be modified for decades, despite having employed different therapeutic strategies.

This situation has changed in the last decade due to the introduction of chemoimmunotherapy, which is already reflected in some population statistics [16]. The median survival has been increased up to 14 years and progression-free survival up to 5 years [17]. These increases in the patients´ survival have been observed since 2003 upon the introduction of the anti-CD20 monoclonal antibody, rituximab (R), in the chemotherapy regimens.

The purpose of the present study was to assess the clinical outcome of patients with FL included in a Spanish registry by the Spanish Lymphoma Oncology Group (GOTEL). This database is part of a prospective registry of all new lymphoma cases, regardless of their histological subtype, which tries to ascertain a possible clinical impact of different therapeutic strategies introduced in the last decades.

## Methods

### Patients

The study was performed as a prospective multicenter study [18]. It was conducted in compliance with the Declaration of Helsinki, and was approved by Puerta de Hierro-Majadahonda Ethics Committee.

Patients referred to the Oncology Department of 18 Spanish hospitals between January 1980 and December 2013, diagnosed with FL were included. Information concerning demographic and clinical-pathological features of each patient as well as prognostic factors, type of treatment and treatment outcome was collected. Patients were staged according to the Ann Arbor system, the FLIPI was calculated, and systemic symptoms were regarded as present when the patient had unexplained fever, night sweats, or weight loss of >10% of initial body weight. Treatment information such as type of therapy (radiation therapy, chemotherapy, combined therapy, or no therapy), response to therapy, and the patient’s survival status, was collected and was assessed in months.

All patients included required a certain diagnosis of follicular lymphoma and were staged according to the Ann Arbor system. Treatment information had to be fully documented, including treatment modality, and initial and final doses. Patients who did not meet any of these criteria were excluded.

The authors served as the advisory board for this study, and participated in all phases of the study, including protocol design, data collection and analysis, and consideration of participating sites.

### Statistical analysis

Overall survival was the end point of all statistical analyses. Survival rates and corresponding standard errors were estimated using Kaplan and Meier estimators. Survival curves were compared applying the log-rank test.

According to the external evaluator’s criteria and due to a lack of sufficient diagnostic, survival or follow up data, 104 patients out of the 1178 patients diagnosed with FL were excluded from the analysis. Therefore we reviewed 1074 patients treated in our country, investigating the use of R and exploring the association between this treatment and survival compared with other patients treated without R in first line or watchful waiting approach in our series with long-follow, and with a median survival of 234 months.

Statistical analyses were performed firstly in all patients and secondly in the treatment group, where efficacy endpoints were analyzed. Overall survival times were estimated with the Kaplan-Meier method and compared with a two-sided log-rank test. Univariate and multivariate Cox regression analysis were used to assess the association between each potential prognostic factor and overall survival and calculate the relative risk (RR). All analyses were two-sided with a 5% significance level and were performed with SPSS version 19 and STATA version 12.

Death hazard risk (HR) according to year of treatment and to age upon diagnosis was calculated. Correlation between group and HR was obtained with the Cox model.

## Results

### Patient characteristics

A total of 1178 patients diagnosed with grade I-IIIa FL between January 1980 and December 2013, in the Oncology Department of 18 Spanish hospitals, were enrolled in the FL Registry, a prospective registry promoted by GOTEL (Spanish Lymphoma Oncology Group) that includes all new lymphoma cases, regardless of their histological subtype. All diagnoses were confirmed by a study hematopathologist. Exclusion criteria were: initial diagnosis of grade IIIb, primary cutaneous FL and HIV positive.

According to the external evaluator´s criteria and due to a lack of sufficient diagnostic, survival or follow up data, 104 patients out of the 1178 patients diagnosed with LF were excluded from the analysis.

The median follow up in the entire series was 54.9 months (1–365.0), 63.7 months (0.1–365.3) only considering patients alive free of disease, 43.7 months (0.1–365.4) for patients alive with the disease, and 32.5 months (0.4–228.4) for those patients who died.

**Fig 1:**
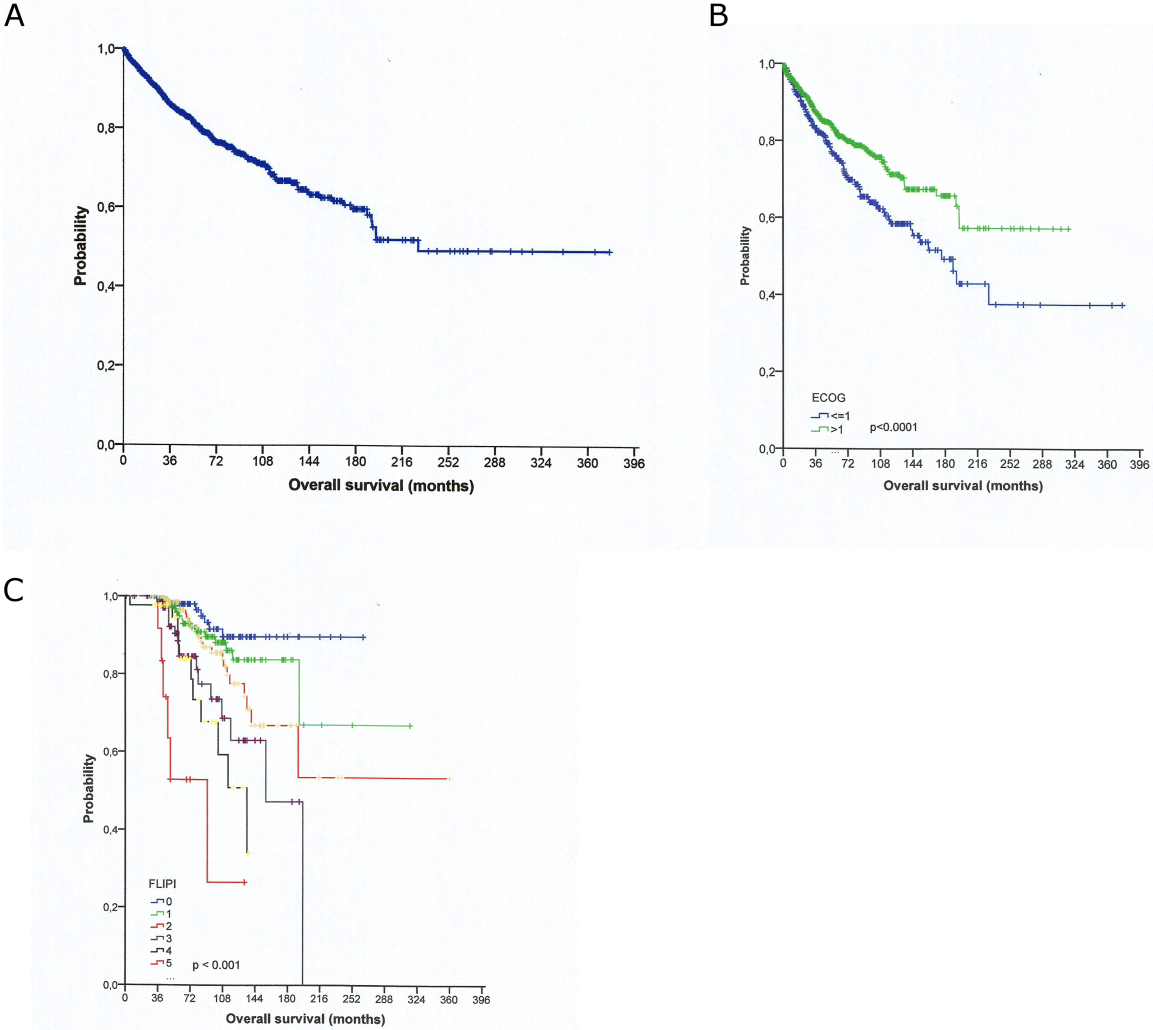
Overall survival (in months) according to performance status ECOG (B) and FLIPI score at diagnosis (C) for all patients (n = 1074).

The main clinical variables registered at the time of diagnosis with potential prognostic relevance are reported in Table 1 and the median follow-up of surviving patients and the OS of these patients are shown in Fig 1.

**Table 1:** Patient characteristics.

Clinical Variables		N
**Sex**	Male	506
	Female	568
**Grade**	I	341
	II	316
	III	129
	IIIa	131
	IIIb	23
	Centrofollicular cutaneous variant	12
	Centrofollicular diffuse variant	50
	NA	72
**Origin**	Nodal	907
	Extranodal	167
**Ann Arbor stage**	I	141
	II	157
	III	273
	IV	500
	NA	3
**ECOG**	0	609
	1	366
	2	68
	3	25
	4	4
	NA	2
**Bone marrow involvement**	No	654
	Yes	417
	NA	3
**B Symptoms**	No	847
	Yes	224
	NA	3
**Bulky mass**	No	820
	Yes	254
**Number of extranodal localizations**	0	602
	1	357
	2	115
**HIV**	No	1072
	Yes	2
**Treatment**	CT with anthracyclines	164
	CT w/o anthracyclines	98
	CT with anthracyclines and R	633
	CT w/o anthracyclines or R	110
	R monotherapy	37
	Cx	13
	Observation	19
**RT**	No	826
	Yes	248
**ASCT**	No	1017
	Yes	57
**Transformation**	No	1040
	Yes	34
**Type of transformation**	Diffuse large B cell	20
	MALT	1
	Hodgkin´s Lymphoma	1
	Mantle B cells lymphoma	1
	Follicular lymphoma grade 3	2
	Burkitt like lymphoma	2
	Angiocentric high grade peripheral T cells lymphoma	1
	High grade follicular lymphoma	1
	Unknown	5
**Cause of death**	Primary tumour	135
	Secondary tumour	21
	Other	68
**Hb**	<12	257
	>12	817
**LDH**	average	812
	high	362
**FLIPI**	0	171
	1	279
	2	337
	3	174
	4	88
	5	25
**B2microglobulin**	average	644
	high	345
	NA	85

CT: chemotherapy; R: rituximab; ECOG: Eastern Cooperative Oncology Group; HIV: human immunodeficiency virus; FLIPI: Follicular Lymphoma International Prognostic Index; Hb: hemoglobin; LDH: lactate dehydrogenase; ASCT: autologous stem cell transplantation; RT: radiotherapy; Cx: surgery

### Follow-up and survival

**Fig 2:**
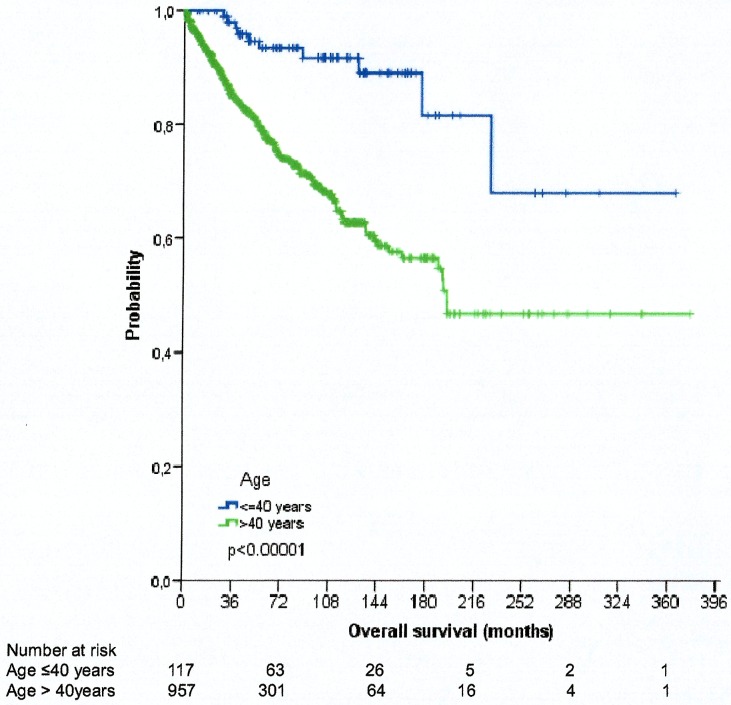
Kaplan-Meier estimates of survival (in years) according to age (over or under 40 years of age).

Significant variables in univariate analysis (Table 2) for OS in our series were incorporated in a multivariate model (Table 3). The multivariate analysis in our series shows greater significance at 60 years of age, as well as performance status (PS), transformation and FLIPI. A cutpoint at 40 years of age was also analyzed to estimate an influence in survival. Our series shows an increase in OS in patients under 40 years old without reaching the median survival, and of 16.3 years in patients over 40 years old, with significant statistical difference (*p* <0.00001) between the curves of OS (Fig 2).

**Table 2:** Results of univariate analysis of different prognostic factors in the whole population of 1074 patients with follicular lymphoma (FL). The table shows the statistically significant prognostic factors.

Overall Survival	N	Median (95% CI)	*%*	*p*
**Age >60**	455	117,2(85.2–149.1)	38	<0.0001
**Ann Arbor stage >II**	730	192.1(148.7–235.5)	85,9	0.003
**ECOG >1**	671	NR	5,1	<0.0001
**Bone marrow involvement**	387	152.1(117.2–187.1)	38,6	0.006
**B symptoms (NO)**	767	NR	81,5	0.02
**Nodal sites>4**	426	176.1(149.9–202.3)	10,4	0.03
**Bulky mass (YES)**	239	170.4(118.2–222.7)	20,3	0.02
**Extranodal sites (=1)**	334	192.7(—)	32,1	0.002
**RT (NO)**	782	NR	69,8	0.001
**ASCT (NO)**	952	228.4(—)	91,8	0.07
**Transformation (NO)**	974	NR	95,8	0.003
**Hb<12**	235	192.1(—)	76,6	0.02
**normal LDH**	757	NR	76,8	<0.0001
**FLIPI 0**	157	NR	20,5	<0.0001
**β2-microglobulin (normal)**	598	NR	71,7	<0.0001
**Treatment with anthracyclines**	199	192.1(113.5–270.7)	17,7	<0.0001

RT: radiotherapy; ECOG: Eastern Cooperative Oncology Group; FLIPI: Follicular Lymphoma International Prognostic Index; Hb: hemoglobin; LDH: lactate dehydrogenase; ASCT: autologous stem cell transplantation; NR: not representative.

**Table 3:** Multivariate analysis: Characteristics associated with overall survival.

Variables	HR (95% CI)	p
**ECOG**	1 ref.	
**ECOG (1)**	1.8(1.3–2.5)	<0.0001
**ECOG (2)**	3.8(2.5–5.8)	<0.0001
**ECOG (3)**	8.3(4.6–14.8)	<0.0001
**ECOG (4)**	15.5(4.7–51.1)	<0.0001
**B Symptoms**	1.4(1.1–1.9)	0.015
**Transformation**	2.6(1.5–4.5)	<0.0001
**FLIPI**	1 ref.	
**FLIPI 1**	2.2(1.1–4.3)	0.03
**FLIPI 2**	2.4(1.2–4.9)	0.01
**FLIPI 3**	3.6(1.8–7.5)	0.001
**FLIPI 4**	3.5(1.6–7.5)	0.001
**FLIPI 5**	4.1(1.6–10.4)	0.001
**Age>60**	1.9(1.5–2.7)	<0.0001

ECOG: Eastern Cooperative Oncology Group; FLIPI: Follicular Lymphoma International Prognostic Index

**Fig 3:**
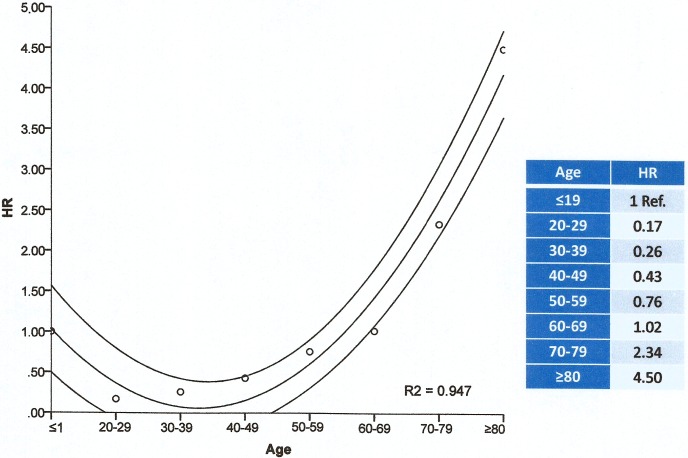
Correlation between group of age and HR value, obtained by Cox model. HR indicates hazard risk and R^2^explains the great correlation between HR and age, with less than a 5% error.

Taking as reference group the young adults group, aged 20–29 years, the risk increases in every group, as shown in Table 4. When calculating HR in Cox model, the reference value always corresponds to minimum risk group, aged ≤19 years, which is why HR increases from 50 years old. Correlation between group of age and HR obtained with Cox model is presented in Fig 3. There is no linear correlation with age, it’s a quadratic correlation. R^2^ shows a great correlation between HR and age, being 94.7% the variability of HR explained by age, with less than a 5% error.

**Table 4:** Age distribution at time of diagnosis of follicular lymphoma for all patients. Correlation between age, mortality, and risk of death, taking as reference group the group aged 20–29 years.

Age	Alive	Dead	N	RR	OR	HR	*p*
**≤29**	20	1	21	0.05	1	Ref.	
**30–39**	64	7	71	0.09	1.8	0.47	*0*.*54*
**40–49**	122	12	134	0.09	1.8	0.99	*0*.*99*
**50–59**	128	22	150	0.15	3	1.87	*0*.*54*
**60–69**	128	28	156	0.18	3.6	3.23	*0*.*25*
**70–79**	52	30	82	0.36	7.2	7.04	*0*.*06*
**≥80**	10	3	13	0.23	4.6	5.69	*0*.*15*

RR: relative risk (of death); OR: overall risk; HR: hazard risk.

An analysis of patients who are still alive beyond 10 years upon diagnosis was conducted, to fully assess the prognostic factors that condition this group. In a group of 166 patients who are still alive after more than 10 years of follow-up, 118 of them (73%) are free of evident clinical disease.

The following variables were not significant: gender, number of nodal areas affected, bone marrow involved or not, lactate dehydrogenase (LDH) levels, nodal or extranodal origin, presence of bulky mass, hemoglobin or transformation to a more aggressive lymphoma. However, stage<II (*p* <0.03), age under 60 years (*p* <0.0001), low FLIPI (*p* <0.002), average B2 microglobulin (*p* <0.005), no B symptoms upon diagnosis (*p* <0.02), PS 0–1 (*p* <0.03) and combined treatment with anthracyclines and R were the variables that were significantly associated with survival at 10 years (*p* <0.0001) (Table 5).

**Table 5:** Variables significantly associated with survival at 10 years.

	N	%	*p value*
**Ann Arbor Stage<II**	44	33.3	*0*.*03*
**Age<60**	96	72.7	*0*.*0001*
**Low FLIPI**	71	53.8	*0*.*002*
**Normal β2 microglobulin**	89	73.6	*0*.*05*
**No B symptoms**	115	87.1	*0*.*02*
**PS 0–1**	129	97.7	*0*.*03*
**Treatment CT+R**	45	39.1	*0*.*001*
**R monotherapy**	60	45.5	*<0*.*0001*

**Fig 4:**
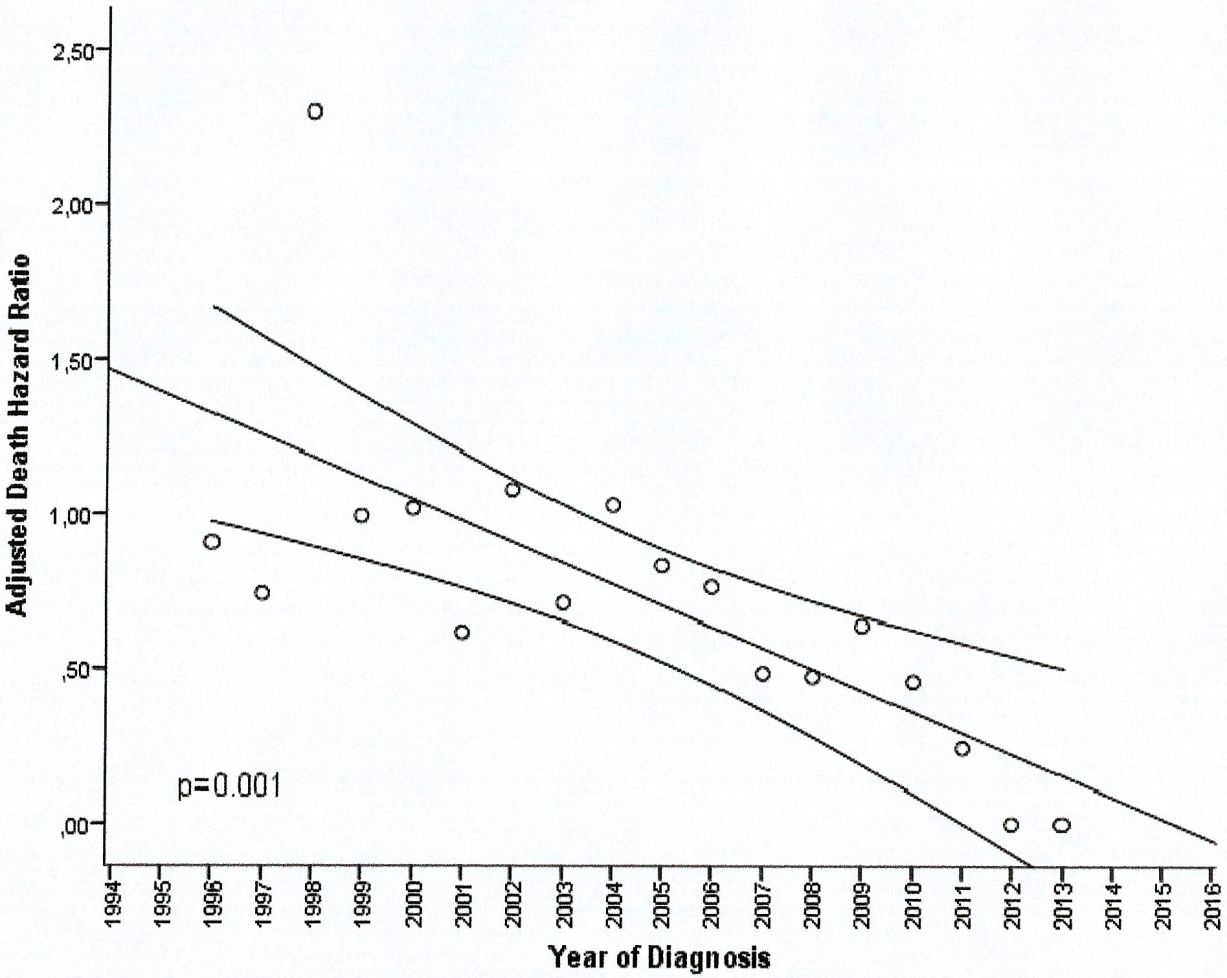
Adjusted death hazard rate to year of diagnosis for the Spanish Lymphoma Study.

CT: chemotherapy; R: rituximab; FLIPI: Follicular Lymphoma International Prognostic Index; PS: performance status;

The HR of death among patients has been declining progressively over the years as can be observed in Fig 4.

## Discussion

Several prognostic factors have been identified in an effort to predict the outcome in patients with FL, including FLIPI, which divides FL cases into three groups with distinct survival probabilities. FLIPI is an internationally validated prognostic index, which provides some hope for improved categorization for patients in clinical studies. Unfortunately, it is solely based on demographic and clinical data and is somehow limited. Alternatively, prognostic models that incorporate biological information, such as those based on gene expression profiles, are potentially much more powerful, although they require validation. Still, and despite such limitations, progress has been made and improvements during the past decade in survival of FL patients have been repeatedly demonstrated in randomized studies and meta-analyses.

In our series, we found significant difference between low/intermediate (<3), and high-risk FLIPI groups (≥3) in terms of OS. Although currently the FLIP2 score is used, we were unable to apply this type of score in our cases due to incomplete availability of some clinical and biological parameters, which were not routinely collected at the time of diagnosis.

The prognostic factors in our series are similar to other series [19, 20, 21] and have already been mentioned: age, B symptoms, stage and tumor burden, extent of bone marrow infiltration, infiltration of specific organs, levels of LDH and β2 microglobulin.

Also, we investigated alternative cutpoints of age other than the traditional 60 years. Thereby, our series shows an increase in overall survival in patients under 40 years of age, without reaching the median survival and those over 40 years of age of 16.3 years, with great statistical difference (*p* <0.00001) between the curves of overall survival. These results are similar to those obtained in a recent study at Princess Margaret Hospital on 61 patients under 40 years of age, showing that the classic cutpoint at 60 years might not be entirely adequate; an earlier age may reflect better prognostic factors and later ages may serve to identify causes of competitive mortality. We looked into this aspect, and our findings suggest that age gives an increased risk of death regardless of the cut point that we use. As a matter of fact, a comparison of clinical features at diagnosis between patients ≤40 and >40 years was carried out in a European series of 1002 patients from 4 different institutions indicated that prognostic factors are useful in the whole population of patients with FL and also apply to younger patients, and that lymphoma-specific survival is similar in young adults and in patients aged 40–60 [22].

The median OS of patients diagnosed with FL is over 20 years in our series. We observed a constant decrease in the HR of death as the years have passed, showing a best prognosis for these patients. Of interest, we wanted to see if a percentage of PFS patients beyond 10 years were sustained. The skill of the therapeutic group seems unlikely to be the explanation of these results, since it is a multicenter study, and also with one of the largest published series that gives a clear consistency to the results. Our data support the initial combined treatment with R and anthracyclines could be considered key factors versus observation.

In 2012 a data analysis from the F2 Study Registry from the International Follicular Lymphoma Prognostic Factor was published. Data from this cohort was compared to patients within the same study but initially treated with regimens that would contain R, in order to know if an initial expectant attitude could influence the effectiveness of these treatments. The 5-year survival was similar in both groups, 87% in the group of patients under observation versus 88% in the group of patients initially treated, concluding that in the R era the strategy of "wait and watch" remains valid for patients with favorable prognostic factors and low-grade tumors (GELF criteria) [23, 24].

First line treatment with R was introduced in Spain only since 2004. However, many patients may have received this treatment during the course of the disease and benefit from it. It seems that the addition of R to chemotherapy schemes has managed to modify the OS of these patients.

Aiming to clarify it, two phase III studies were launched; the National Lympho Care Study [25] and FOLL-05 [26]. No difference was observed in either of them between R-CHOP (cyclophosphamide, doxorubicine, vincristine, prednisone), R-CVP (cyclophosphamide, vincristine, prednisone) and R-FM (fludarabine and mitoxantrone), in terms of OS or progression-free survival. Increased time to progression (27 months vs 7 months) and longer survival at 4 years (83% vs 77%) was also observed [27].

We observed an increase in OS of patients over the years and diagnostic times, which are identifiable in all age and sex groups, including advanced stages. This finding is consistent with data from American [28, 29] and European [30] studies, ours being the largest study of all the ones published in our continent that can be related to the introduction of R. It has been speculated whether the improvements achieved in support care or even high doses of chemotherapy may have influenced this.

According to our results, especially taking into account the study of patients alive for more than 10 years, we believe that the weight of the introduction of R in a young population, associated with chemotherapy, has given these high rates of survival in an unselected population. The median OS of patients diagnosed of FL is over 20 years in our series, which suggest that increase in survival might be due to the use of anthracyclines, R and radiotherapy.

The development of national registries such as the Spanish Follicular Lymphoma Registry, promoted by GOTEL, help us identify the clinical-pathological characteristics of the patients in our area and therefore try to develop the best treatment program for our patients, improving the effectiveness of our clinical practice.
